# Deletions of *pfhrp2* and *pfhrp3* genes of *Plasmodium falciparum* from Honduras, Guatemala and Nicaragua

**DOI:** 10.1186/s12936-018-2470-7

**Published:** 2018-08-31

**Authors:** Gustavo Fontecha, Rosa E. Mejía, Engels Banegas, Maria Paz Ade, Lisandro Mendoza, Bryan Ortiz, Isaac Sabillón, Gerardo Alvarado, Gabriela Matamoros, Alejandra Pinto

**Affiliations:** 1 Microbiology Research Institute, Universidad Nacional Autonoma de Honduras, Tegucigalpa, Honduras; 2 Panamerican Health Organization, Tegucigalpa, Honduras; 3 National Department of Surveillance, Ministry of Health, Tegucigalpa, Honduras; 4 Panamerican Health Organization, Washington, USA

**Keywords:** Malaria, Central America, *Plasmodium falciparum*, RDT, *pfhrp2*, *pfhrp3*

## Abstract

**Background:**

Malaria remains a public health problem in some countries of Central America. Rapid diagnostic tests (RDTs) are one of the most useful tools to assist in the diagnosis of malaria in remote areas. Since its introduction, a wide variety of RDTs have been developed for the detection of different parasite antigens. PfHRP2 is the most targeted antigen for the detection of *Plasmodium falciparum* infections. Genetic mutations and gene deletions are important factors influencing or affecting the performance of rapid diagnostic tests.

**Methods:**

In order to demonstrate the presence or absence of the *pfhrp2* and *pfhrp3* genes and their flanking regions, a total of 128 blood samples from patients with *P. falciparum* infection from three Central American countries were analysed through nested or semi-nested PCR approaches.

**Results:**

In total, 25.8 and 91.4% of the isolates lacked the region located between exon 1 and exon 2 of *pfhrp2* and *pfhrp3* genes, respectively. Parasites from the three countries showed deletions of one or both genes. The highest proportion of *pfhrp2* deletions was found in Nicaragua while the isolates from Guatemala revealed the lowest number of *pfhrp2* deletions. Parasites collected from Honduras showed the highest proportion of *phfrp3* absence (96.2%). Twenty-one percent of isolates were double negative mutants for the exon 1–2 segment of both genes, and 6.3% of isolates lacked the full-length coding region of both genes.

**Conclusions:**

This study provides molecular evidence of the existence of *P. falciparum* isolates lacking the *pfhrp2* and *pfhrp3* genes, and their flanking regions, in Honduras, Guatemala and Nicaragua. This finding could hinder progress in the control and elimination of malaria in Central America. Continuous evaluation of RDTs and molecular surveillance would be recommended.

## Background

Malaria remains a public health problem for most tropical countries. However, there are notable differences in the incidence of malaria between geographic regions. Within the Central American sub region, two countries (Costa Rica and El Salvador) reported fewer than 15 indigenous malaria cases in 2016, and, according to the World Health Organization, are projected to eliminate malaria by 2020. In Nicaragua, Guatemala and Honduras however, a total of 15,476 cases were reported in 2016, representing the largest burden in Central America. Although the prevalence of malaria is decreasing in this region, Panama and Nicaragua showed an increase in case incidence between 2010 and 2016. Approximately, 90% of malaria cases in Nicaragua and Honduras are due to *Plasmodium vivax*, and the remaining 10% to *Plasmodium falciparum.* Guatemala, on the other hand, has been reporting fewer than 10 cases of falciparum malaria in recent years [1].

As this sub-region of the Americas approaches the goal of eliminating malaria by the year 2030 [2], it is necessary to include more intensive strategies of prevention, timely diagnosis and treatment of infections, especially in areas with low social development, where appropriate health infrastructure is not available. Rapid diagnostic tests (RDTs) based on immunochromatography are one of the most useful tools to assist in the diagnosis of malaria in the absence of good quality microscopy services. Since their introduction, a wide variety of RDTs have been developed for the detection of different parasite antigens [3]. Two antigens (e.g. aldolase, pLDH) are produced by all *Plasmodium* species (pan-specific tests), but the PfHRP2 antigen (Histidine-Rich Protein-2) is produced only by *P. falciparum*. PfHRP2 is an abundant and heat stable antigen, which makes it a highly sensitive target for the diagnosis of falciparum malaria [3, 4].

PfHRP2 is a non-essential protein encoded by *pfhrp2* gene, located on chromosome 8 of *P. falciparum,* and *pfhrp3* is an structural homologue of *pfhrp2* [5], which is located on chromosome 13. Both antigens cross-react when detected by some PfHRP2-based RDTs [6]. A retrospective study carried out with 128 samples collected in Iquitos reported that 41 and 70% lacked the *pfhrp2* or *pfhrp3* genes, respectively, and 21.6% of parasites lacked both genes [7]. Such findings represent an emerging challenge for the diagnosis and control of malaria, especially since several worldwide studies report parasite isolates that present double deletions for these genes [8–14]. All this information has been conveniently summarized by WHO in a website [15] that allows to perceive graphically the spread of parasite isolates for which the PfHRP2-based RDTs could no longer be effective. For this reason, WHO has prioritized efforts to address the problem posed by parasites with these deletions [16, 17], and proposes that the analysis of archival samples could be useful to determine the existence and geographic distribution of the double-negative populations of the parasite [16].

The only study published on this topic in Central America dates from 2015, in which samples from Honduras collected between 2008 and 2009 were analysed through a molecular approach [18]. These authors do not report isolates with double-deletions but found a high proportion of *pfhrp3*-negative parasites and suggested that PfHRP2-based RDTs could continue to be used in Honduras, but at the same time they propose further studies including samples from a wider geographical area. Thus, this study aims to be an update of the topic for the Central American sub-region, analysing populations of the parasite from Guatemala, Nicaragua and Honduras.

## Methods

**Fig. 1:**
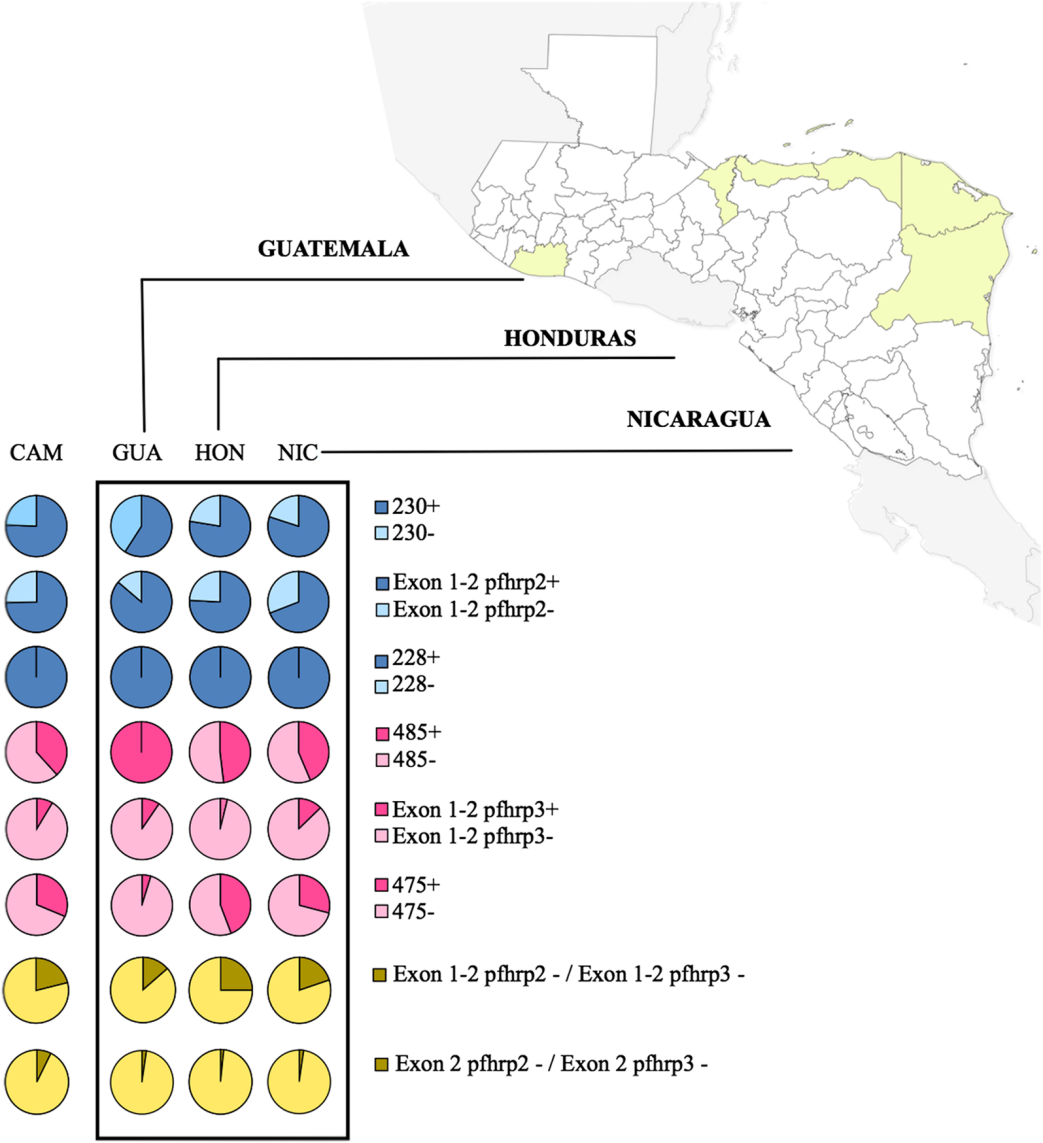
Proportion of deletions in *pfhrp2* and *pfhrp3* genes and flanking regions in Plasmodium falciparum isolates from Central America (CAM). The pie charts illustrate the proportion of parasite isolates with or without deletions by country: Guatemala, Honduras and Nicaragua. The departments where the samples were collected are coloured in yellow

The aim of the study was to evaluate the presence of the *pfhrp2* and *pfhrp3* genes and their flanking regions in 128 falciparum malaria samples from Central America, previously diagnosed by microscopy. Twenty-one samples were collected from Guatemala and 55 samples from Nicaragua, during 2015, and 52 samples from Honduras were collected during 2011 (n = 10), 2012 (n = 11) and 2017 (n = 31). Samples from Guatemala were collected from the department of Escuintla. In Nicaragua the samples came from the North Atlantic Autonomous Region (RAAN), while in Honduras the majority of samples came from the department of Gracias a Dios and less than 10% were obtained from five other departments (Colón, Atlántida, Cortés and Islas de la Bahía) (Fig. 1). The samples were collected for routine diagnosis and malaria surveillance in the three countries in different endemic localities. The criteria for the selection of samples was by convenience. The Ministries of Health of each country selected a random percentage of positive samples for their analysis. Patients’ informed consent was requested for diagnostic procedures. After confirming the diagnosis of falciparum malaria by microscopic observation of the parasite, a blood sample was collected on Whatman™ filter paper (GE Healthcare Bio-Sciences Corp, NJ, USA) for routine chloroquine resistance surveillance through a molecular approach [19, 20]. Parasite densities were determined for most samples. Samples with less than 10 parasites/100 white blood cells (WBC) were considered as low density. A moderate density parasitaemia included samples with 10–50 parasites/100 WBC. Samples with more than 50 parasites/100 WBC were considered as high density parasitaemia.

Those blood samples were used for the analysis of *pfhrp2* and *pfhrp3* deletions. DNA was extracted using a Chelex-100 based method [21]. In order to assess the quality of the extracted DNA, a region of the human beta-globin gene was amplified by conventional PCR [22]. The microscopic diagnosis of the parasite species was confirmed through a species-specific PCR approach using the primers AL7178/AL7142 and AL7175/AL7074 according to previous reports [23, 24]. The amplification of parasite sequences was indicative of a good-quality DNA that would allow the detection of target sequences for the genes *pfhrp2*, *pfhrp3* and flanking sequences. In order to rule out the possibility of false negative results attributable to poor DNA quality or insufficient amounts of the parasite’s genome, the amplification of the single copy gene *pfmsp*1 in those samples showing a double deletion in partial sequences of *pfhrp2* and *pfhrp3* genes was performed. The amplification procedures were carried out through a nested PCR according to previous reports [25, 26].

**Fig. 2:**
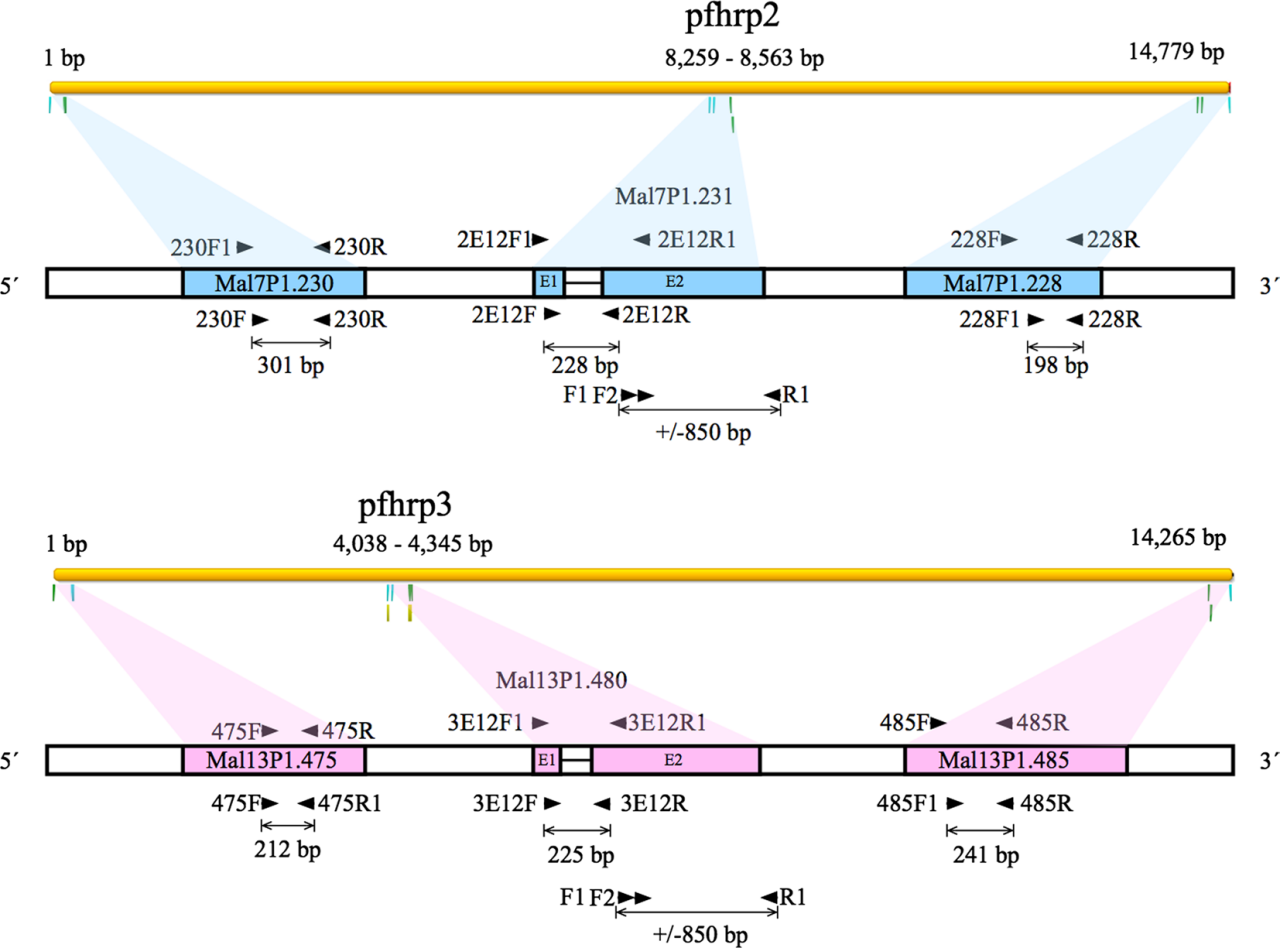
Scheme of the *pfhrp2* and *pfhrp3* genes and their flanking sequences. The black arrows indicate the names and targets of the primers used to amplify each region, as well as the size of the amplicons. The parasite strain used for the in silico analysis was 3D7 (GenBank Accession Number: AL844507.3)

In order to detect the presence or absence of a partial coding region between exons 1 and 2 of the genes *pfhrp2* and *pfhrp3*, and the flanking regions upstream and downstream of each gene, 6 nested or semi-nested PCR reactions were performed as outlined in Fig. 2 [18]. Primers’ list, sequences, annealing temperatures, and amplicon sizes are detailed in Table 1. Briefly, each reaction was carried out in a volume of 25 μl composed of 12.5 μl of 2X Master mix (Promega Corp.), 1.0 μl of each primer at a concentration of 10 μM and 2.0 μl of genomic DNA. Reactions of both the primary and nested PCR were carried out by an initial denaturation temperature at 95 °C for 5 min, followed by 35 cycles of 95 °C for 1 min, an annealing step for 1 min, 72 °C for 1 min; and a final extension step at 72 °C for 5 min. A second PCR was performed after each initial reaction, including 1 μl of the first PCR product. Two positive and negative controls were used within each experiment. All negative results were amplified a second time. If a positive result was obtained, it was considered positive. The coding regions of those samples with double deletions (*pfhrp2* and *pfhrp3* negative) were amplified a third time.

**Table 1:** Primer sequences and amplification conditions of *pfhrp2* and *pfhrp3* genes, and their respective flanking sequences

Target sequence	Reaction	Primer	Primer sequence	Annealing temp (°C)	Amplicon size (bp)
pfhrp2 UPSTREAM PF3D7_0831900, (MAL7P1.230)	Primary	230F1	5′GATATCATTAGAAAACAAGAGCTTAG3′	63	301
		230R	5′TATCCAATCCTTCCTTTGCAACACC3′		
	Semi-nested	230F	5′TATGAACGCAATTTAAGTGAGGCAG3′	65	
		230R	5′TATCCAATCCTTCCTTTGCAACACC3′		
pfhrp2 Exon 1–2, PF3D7_0831800	Primary	2E12F1	5′GGTTTCCTTCTCAAAAAATAAAG3′	55	228
		2E12R1	5′TCTACATGTGCTTGAGTTTCG3′		
	Nested	2E12F	5′GTATTATCCGCTGCCGTTTTTGCC3′	62	
		2E12R	5′CTACACAAGTTATTATTAAATGCGGAA3′		
pfhrp2 DOWNSTREAM PF3D7_0831700, (MAL7P1.228)	Primary	228F	5′AGACAAGCTACCAAAGATGCAGGTG3′	60	198
		228R	5′TAAATGTGTATCTCCTGAGGTAGC3′		
	Semi-nested	228F1	5′CCATTGCTGGTTTAAATGTTTTAAG3′	63	
		228R	5′TAAATGTGTATCTCCTGAGGTAGC3′		
pfhrp3 DOWNSTREAM PF3D7_1372100, (MAL13P1.485)	Primary	485F	5′TTGAGTGCAATGATGAGTGGAG3′	60	241
		485R	5′AAATCATTTCCTTTTACACTAGTGC3′		
	Semi-nested	485F1	5′GTTACTACATTAGTGATGCATTC3′	59	
		485R	5′AAATCATTTCCTTTTACACTAGTGC3′		
pfhrp3 Exon 1–2, PF3D7_1372200	Primary	3E12F1	5′GGTTTCCTTCTCAAAAAATAAAA3′	53	225
		3E12R1	5′CCTGCATGTGCTTGACTTTA3′		
	Nested	3E12F	5′ATATTATCGCTGCCGTTTTTGCT3′	62	
		3E12R	5′CTAAACAAGTTATTGTTAAATTCGGAG3′		
pfhrp3 UPSTREAM PF3D7_1372400, (MAL13P1.475)	Primary	475F	5′TTCATGAGTAGATGTCCTAGGAG3′	55	212
		475R	5′TCGTACAATTCATCATACTCACC3′		
	Semi-nested	475F	5′TTCATGAGTAGATGTCCTAGGAG3′	61	
		475R1	5′GGATGTTTCGACATTTTCGTCG3′		
pfhrp2 Exon 2	Primary	Pfhrp2F1	5′CAAAAGGACTTAATTTAAATAAGAG3′	55	600–950
		Pfhrp2R1	5′AATAAATTTAATGGCGTAGGCA3′		
	Semi-nested	Pfhrp2F2	5′ATTATTACACGAAACTCAAGCAC3′	55	
		Pfhrp2R1	5′AATAAATTTAATGGCGTAGGCA3′		
pfhrp3 Exon 2	Primary	Pfhrp3F1	5′AATGCAAAAGGACTTAATTC3′	55	600–950
		Pfhrp3R1	5′TGGTGTAAGTGATGCGTAGT3′		
	Semi-nested	Pfhrp3F2	5′AAATAAGAGATTATTACACGAAAG3′	55	
		Pfhrp3R1	5′TGGTGTAAGTGATGCGTAGT3′		

To confirm the complete lack of the *pfhrp2* and *pfhrp3* genes, all samples that did not amplify the exon 1–2 segment of both genes were amplified by a semi-nested PCR targeting the exon 2. Both reactions were carried out in a 50 μl volume and included 25 μl of 2× Master mix (Promega Corp.), 2.0 μl of each 10 μM primer (F1/R1 in the first round and F2/R1 in the second round), and 1.0 μl of DNA. Reactions were carried out by an initial denaturation temperature at 94 °C for 10 min, followed by 35 and 37 cycles respectively of 94 °C for 50 s, an annealing step for 50 s, 72 °C for 1 min, and a final extension step at 72 °C for 10 min (Table 1). The expected PCR product ranged between 600 and 950 bp.

## Results

A total of 128 blood samples from patients with *P. falciparum* infection were diagnosed by microscopy in three Central American countries, which is the gold standard method for malaria. All the samples were confirmed to be falciparum infections through a molecular approach. 50.6% of the samples showed high parasitic density, 27.8% were moderate and 21.5% were low. Samples from the three countries showed heterogeneous parasitic density with no correlation between geographical origin and parasitic density. Coding regions of the *pfhrp2* and *pfhrp3* genes and the corresponding upstream and downstream flanking regions were amplified by nested PCR. Overall, 25.8% of the isolates were negative for the partial coding region between exon 1–2 (intron 1) of *pfhrp2* and 91.4% of the isolates lacked the homologous region of *pfhrp3* (Table 2). Parasites from the three countries showed deletions of one or both gene regions. The highest proportion of exon 1–2 *pfhrp2* deletions was found in Nicaragua (30.9%) while the isolates from Guatemala revealed the lowest number of deletions (14.3%). Parasites collected from Honduras showed the highest proportion of exon 1–2 *phfrp3* deletion (96.2%).

**Table 2:** Number and percentages of samples showing deletions of the pfhrp2 and pfhrp3 partial sequences (exon 1–2) by country and year of collection

Country	Year of collection	Exon 1–2 pfhrp2+	Exon 1–2 pfhrp2−	Exon 1–2 pfhrp3+	Exon 1–2 pfhrp3−	Total
Guatemala	2015	18 (85.7%)	3 (14.3%)	2 (9.5%)	19 (90.5%)	21 (16.4%)
Nicaragua	2015	38 (69.1%)	17 (30.9%)	7 (12.7%)	48 (87.3%)	55 (43%)
Honduras	2011	6 (60%)	4 (40%)	–	10 (100%)	10 (7.8%)
Honduras	2012	11 (100%)	–	2 (18.2%)	9 (81.8%)	11 (8.6%)
Honduras	2017	22 (71%)	9 (29%)	–	31 (100%)	31 (24.2%)
	Total	95 (74.2%)	33 (25.8%)	11 (8.6%)	117 (91.4%)	128 (100%)

A relevant result was the finding of 27 (21%) double negative isolates (*pfhrp2* negative and *pfhrp3* negative). Most of those double negative parasites were detected in Honduras 13/52 (25%), followed by Nicaragua 11/55 (20%) and Guatemala 3/21 (14.3%). No significant differences were found in the frequency of the deletions between the three different years of collection in Honduras. The *pfmsp*-1 gene was successfully amplified in the 27 double negative isolates, showing a distribution of 52% for the K1 genotype and 48% for MAD20.

Nested PCRs were also carried out to amplify the flanking regions of *pfhrp2* (MAL7P1.230 and MAL7P1.228), and *pfhrp3* (MAL13P1.485 and MAL13P1.475). Most of the isolates (75.8%) revealed the presence of the MAL7P1.230 region, and almost all the isolates (99.2%) successfully amplified the MAL7P1.228 region. On the other hand, more than 62% of isolates seem to have deleted the MAL13P1.485 and MAL13P1.475 regions (Table 3, Fig. 2). When comparing the 6 markers, the most frequently deleted locus was *pfhrp3* (91.4%), followed by MAL13P1.475 (68.8%). All samples amplified properly at least one of the 6 analysed loci, which indicates that the quality and quantity of DNA was sufficient to guarantee reliability in the negative results.

**Table 3:** Number and percentages of samples showing deletions of the pfhrp2 and pfhrp3 genes and their flanking regions by country

MAL7P1.230	pfhrp2 Exon 1–2	MAL7P1.228	Guatemala, n (%)	Nicaragua, n (%)	Honduras, n (%)	Total, n (%)
+	+	+	12 (57.1)	30 (54.5)	33 (63.5)	75 (58.6)
−	+	+	6 (28.6)	8 (14.5)	6 (11.5)	20 (15.6)
+	−	+	–	14 (25.4)	7 (13.5)	21 (16.4)
−	−	+	3 (14.3)	3 (5.5)	5 (9.6)	11 (8.6)
+	−	−	–	–	1 (1.9)	1 (0.8)
		Total	21 (100)	55 (100)	52 (100)	128

MAL13P1.485	pfhrp3 Exon 1–2	MAL13P1.475	Guatemala, n (%)	Nicaragua, n (%)	Honduras, n (%)	Total, n (%)
−	−	−	18 (85.7)	21 (38.2)	23 (44.2)	62 (48.4)
+	−	−	–	15 (27.3)	6 (11.5)	21 (16.4)
+	−	+	–	5 (9.1)	17 (32.7)	22 (17.2)
−	−	+	1 (4.8)	7 (12.7)	4 (7.7)	12 (9.4)
+	+	+	–	4 (7.3)	2 (3.8)	6 (4.7)
−	+	−	2 (9.5)	3 (5.4)	–	5 (3.9)
		Total	21 (100)	55 (100)	52 (100)	128

Five deletion patterns were recorded when analysing the exon 1–2 *pfhrp2* sequence plus the flanking regions, while the loci associated to *pfhrp3* revealed 6 different deletion patterns (Table 3). Most of the samples (58.6%) did not reveal any deletion in the analysed region of the chromosome 8, however < 5% of the samples seem to keep the region of the chromosome 13 intact without deletions. None of the isolates showed a complete lack of the exon 1–2 region of *pfhrp2* gene and both flanking genes, while many samples (48.4%) seem to have deleted the entire *pfhrp3* region.

In order to confirm the deletion of the *pfhrp2* and *pfhrp3* genes, the full length of exon 2 was amplified [27] in those samples that showed a double deletion of the sequence between exon 1 and exon 2. As shown in Table 4, 8 of 27 isolates revealed total absence of the two exons in both genes. The rest of the isolates amplified at least one exon 2 of either of both genes. Isolates with complete deletions of both genes were detected in all three countries.

**Table 4:** Number and percentages of samples showing deletions of pfhrp2 and pfhrp3 sequences (exon 1–2 and exon 2) by country

Exon 1–2 pfhrp2	Exon 2 pfhrp2	Exon 1–2 pfhrp3	Exon 2 pfhrp3	Guatemala, n (%)	Nicaragua, n (%)	Honduras, n (%)	Total, n (%)
−	−	−	−	3 (11.1)	2 (7.4)	3 (11.1)	8 (29.6)
−	−	−	+	0	1 (3.7)	1 (3.7)	2 (7.4)
−	+	−	+	0	6 (22.2)	6 (22.2)	12 (44.4)
−	+	−	+	0	2 (7.4)	3 (11.1)	5 (18.5)
				3 (11.1)	11 (40.7)	13 (48.1)	27 (100)

## Discussion

This is the first report of deletions of the genes *pfhrp2*, *pfhrp3* and their neighbouring regions in *P. falciparum* parasites collected from Guatemala and Nicaragua, and this is also the second report that includes samples collected from Honduras [18]. Motivated by the growing number of similar reports in South America [7–10, 28–30], Asia [11, 12, 31] and Africa [14, 32–36], this study analysed the presence or absence of 6 loci located on chromosomes 8 and 13 of the parasite using archived specimens from Central America [16] with the aim of estimating the frequency of deletions around the genes *pfhrp2* and *pfhrp3*.

Although RDTs are a valuable tool to establish the diagnosis of falciparum malaria, especially in areas where routine microscopic diagnosis is not available, these tests could yield false negative results due to undetectable concentrations of the PfHRP2 antigen in low parasitaemias [37], to the absence of expression of this protein when the parasite has deleted some genomic segments [16] or other causes listed by WHO [17] that should be investigated in each region or country. The main consequence of the deletions of *pfhrp2* and *pfhrp3* genes is the impossibility of establishing an adequate diagnosis of malaria, especially where it is solely based on RDTs that exclusively detect the PfHRP2 protein [7, 38, 39].

Amplification of coding sequences and their flanking regions through a nested-PCR approach is a commonly used method to investigate deletions of *pfhrp2* and *pfhrp3* [18, 27, 38, 40]. Due to the challenge of guaranteeing the absence of a genetic sequence based only on a negative amplification result, some experiments were conducted to achieve the greatest certainty possible. Each negative experiment was repeated up to 3 times. In addition, the single copy gene *pfmsp*-1 was successfully amplified for the samples that yielded a negative result for both gene regions. The results obtained suggest that the absence of amplification does not seem to be due to a low DNA concentration of the parasite, since it was not possible to establish any association between samples with low, moderate or high parasitaemia and absence of amplification for any loci. Also, given that all samples amplified at least one of the 6 analysed loci, these results seem to be reliable and allow to confirm the existence of deletions of the sequences associated with the genes *pfhrp2*, *pfhrp3* and their flanking regions in the natural populations of *P. falciparum* circulating in Central America.

Before this study, there was not enough information of deletions in the *pfhrp2* and *pfhrp3* genes in parasites from Central America [15]. The only study available analysed 68 samples collected between 2008 and 2009, in Puerto Lempira city in the Honduran Moskitia [18]. Those authors found that all samples were positive for *pfhrp2* and its flanking sequences, and therefore PfHRP2-based RDTs could be considered useful in this geographic region at the time. Interestingly, in this study 10 archival samples collected 2 years later (2011) in Honduras showed 40% of isolates with deletion in the *pfhrp2* gene. This could be attributed both to the selective adaptation of the parasite in a short period of time, and to the fact that the samples were collected in some regions of the country that were not represented in the previous study (Colon, Roatan and Gracias a Dios). Abdallah et al. [18] also reported 44% of parasites lacking the *pfhrp3* region and suggested the need to expand the research with samples collected from other geographic regions and for a longer time span. Following that recommendation, and according to the results obtained in this study, the proportion of parasites lacking *pfhrp3* in this study area was higher than that previously reported in Puerto Lempira. This high percentage of *pfhrp3*-negative parasites is in agreement with the large genomic deletions in the chromosome 13 described in the reference line HB3, a parasite isolate collected from Honduras [41].

When analysing all the samples collected from the three Central American countries, 75% of the isolates amplified successfully the *pfhrp2* gene, while only 9% of the isolates were *pfhrp3*-positive. A WHO web mapping tool that tracks 3 of the major biological threats to malaria control and elimination compiled 53 surveys from 7 South American countries regarding *pfhrp2/pfhrp3* deletions [42]. Despite the heterogeneity of all these surveys, there is a general trend indicating that deletions in the *pfhrp2* gene are less frequent than those in the *pfhrp3* gene; which is consistent with the current results. These coincidences could be attributed to similar characteristics in the evolution of the parasites from South and Central America. The high percentage of deletions in the *pfhrp3* gene that are observed in some localities of Colombia [8] and Peru [7] is also of special interest.

Regarding the flanking sequences of the *pfhrp2/pfhrp3* genes, all isolates retained the downstream locus MAL7P1.228, in a similar way to that reported in Eritrea [38, 40], where the MAL13P1.475 region was also the most frequently deleted locus. When comparing the results of this study with those reported previously [18], it is noteworthy that the 4 flanking regions, both of *pfhrp2* and *pfhrp3* now reveal a high number of deletions, which would support the hypothesis of the rapid appearance of deletions in these genetic regions. Nevertheless, one of the most interesting results of this study is the finding of 26 (20.3%) isolates with a double deletion (*pfhrp2*/*pfhrp3* negative for exon 1–2). Some authors have stated that the deletion of those particular genetic sequences is the result of multiple independent events instead of the dispersion of strains originated from a single event [43, 44]. However, once they emerge, the rapid spread of double-negative populations could be caused by the selection of these parasites by the pressure acting on these variants [45], as demonstrated also by stochastic simulation when the use of RDTs is based on the exclusive detection of the PfHRP2 antigen [46]. Despite the reason behind the emergence and spread of this genotypes in Central America, the presence of double-negative parasites in this endemic region has direct implications on the use of RDTs for routine diagnosis, especially in areas where microscopy is not feasible. This new finding could pose a threat to national malaria programmes, mainly due to the risk for patients infected with these parasites not receiving a timely and correct diagnosis and treatment; as well, non-PfHRP2-based RDTs have demonstrated limited sensitivity and heat instability [16].

In the three countries analysed in this study, the Ministries of Health use RDTs based on detection of the PfHRP2 antigen: CareStart™ Malaria HRP2/pLDH(Pf/Pv) Combo (Honduras), SD Bioline MALARIA Ag P.f/P.v (Nicaragua) and CareStart™Malaria RDT Single Kit (Guatemala). According to the World Malaria Report 2017 [1], in 2016 Honduras, Guatemala and Nicaragua performed 14,930, 74,859 and 880 RDTs, respectively, but only Honduras reported positive results by RDT (n = 241). These data represent 8.9, 22.4 and 0.14% of the total number of diagnostic tests when compared to the microscopic approach. Considering that most of the diagnosis of malaria in these three countries is still carried out through microscopy and the prevalence of *P. falciparum* infections is considerably less than *P. vivax*, the impact of a few false negative results derived from RDTs detecting PfHRP2 would be low for the public health. However, although the risk of diagnostic failures due to non-detection of the parasite by RDT could be low, the countries of the region have proposed eliminating malaria by the year 2030, and this goal requires rapid and timely detection of all cases of malaria, especially in the most remote areas. Consequently, it will be necessary to evaluate the performance of those particular RDTs used in Central America and establish a routine molecular surveillance programme [11].

An interesting result of this study is that most of the isolates (17/27) that did not amplify the intron 1 segment of the *pfhrp2* gene seem to have intact the exon 2 (Table 4). A similar phenomenon occurred for 19 of 27 isolates without the intron 1 of *pfhrp3* that successfully amplified the full-length exon 2. A possible explanation for this apparent discrepancy could be the presence of mutations in the primers targets that prevent the amplification of this gene region. However, a more likely hypothesis could be that some parasites suffered breakage points at the end of exon 1 of both genes, as indicated by Cheng et al. [27], which seems to be a relatively common event. With the obtained data it is not possible to assure if the partial or total absence of exon 1 of both genes has consequences for the expression of the PfHRP2 and PfHRP3 antigens, especially because the epitopes relevant for their detection are completely encoded in exon 2 [47] In any case, these results demonstrate the existence of some strains with complete deletions of both genes.

The use of archived samples was a main limitation of the study. Samples were not analysed by serology or RDTs, as recommended by Cheng et al. and supported by the WHO [16, 27, 31]. Therefore, it is not possible to confirm with absolute reliability the diagnostic failure of PfHRP2-based RDTs in Central America. Thus, it will be convenient to carry out further studies comparing the performance of microscopy, RDTs and molecular analysis. As well, sequencing studies of the *pfhrp2* gene searching for polymorphisms that could affect the sensitivity of the RDTs are recommended [12, 14, 48, 49].

## Conclusions

In summary, this study provides molecular evidence of the existence of *P. falciparum* isolates lacking the *pfhrp2* and *pfhrp3* genes, and flanking regions, in Honduras, Guatemala and Nicaragua. This finding could hinder the progress made in the control and elimination of malaria in Central America. From now on, a routine evaluation of the performance of RDTs and molecular surveillance will be required.

## Abbreviations

*pfhrp2* (PfHRP2): *Plasmodium falciparum* histidine-rich protein 2
*pfhrp3* (PfHRP3): *Plasmodium falciparum* histidine-rich protein 3
WHO: World Health Organization
*pfmsp*-1: *Plasmodium falciparum* merozoite surface protein 1
RDT: rapid diagnostic test
